# Increased Circulating ANG II and TNF-α Represents Important Risk Factors in Obese Saudi Adults with Hypertension Irrespective of Diabetic Status and BMI

**DOI:** 10.1371/journal.pone.0051255

**Published:** 2012-12-12

**Authors:** Nasser M. Al-Daghri, Lotfi S. Bindahman, Omar S. Al-Attas, Tahia H. Saleem, Majed S. Alokail, Khalid M. Alkharfy, Hossam M. Draz, Sobhy Yakout, Amany O. Mohamed, Alison L. Harte, Philip G. McTernan

**Affiliations:** 1 Biomarkers Research Program, Department of Biochemistry, College of Science, King Saud University, Riyadh, Saudi Arabia; 2 Center of Excellence in Biotechnology Research, King Saud University, Riyadh, Saudi Arabia; 3 Biochemistry Department, Faculty of Medicine, Assiut University, Assiut, Egypt; 4 Department of Clinical Pharmacy, College of Pharmacy, King Saud University, Riyadh, Saudi Arabia; 5 Department of Biochemistry, National Research Centre, Dokki, Cairo, Egypt; 6 Division of Metabolic and Vascular Health, Warwick Medical School, University of Warwick, CSRL, University Hospitals Coventry and Warwickshire Trust, Walsgrave, Coventry, United Kingdom; University of Hong Kong, China

## Abstract

Central adiposity is a significant determinant of obesity-related hypertension risk, which may arise due to the pathogenic inflammatory nature of the abdominal fat depot. However, the influence of pro-inflammatory adipokines on blood pressure in the obese hypertensive phenotype has not been well established in Saudi subjects. As such, our study investigated whether inflammatory factors may represent useful biomarkers to delineate hypertension risk in a Saudi cohort with and without hypertension and/or diabetes mellitus type 2 (DMT2). Subjects were subdivided into four groups: healthy lean controls (age: 47.9±5.1 yr; BMI: 22.9±2.1 Kg/m^2^), non-hypertensive obese (age: 46.1±5.0 yr; BMI: 33.7±4.2 Kg/m^2^), hypertensive obese (age: 48.6±6.1 yr; BMI: 36.5±7.7 Kg/m^2^) and hypertensive obese with DMT2 (age: 50.8±6.0 yr; BMI: 35.3±6.7 Kg/m^2^). Anthropometric data were collected from all subjects and fasting blood samples were utilized for biochemical analysis. Serum angiotensin II (ANG II) levels were elevated in hypertensive obese (*p*<0.05) and hypertensive obese with DMT2 (*p*<0.001) compared with normotensive controls. Systolic blood pressure was positively associated with BMI (p<0.001), glucose (*p*<0.001), insulin (p<0.05), HOMA-IR (p<0.001), leptin (p<0.01), TNF-α (p<0.001) and ANG II (p<0.05). Associations between ANG II and TNF-α with systolic blood pressure remained significant after controlling for BMI. Additionally CRP (p<0.05), leptin (p<0.001) and leptin/adiponectin ratio (p<0.001) were also significantly associated with the hypertension phenotype. In conclusion our data suggests that circulating pro-inflammatory adipokines, particularly ANG II and, TNF-α, represent important factors associated with a hypertension phenotype and may directly contribute to predicting and exacerbating hypertension risk.

## Introduction

Hypertension is a heterogeneous condition that is positively linked with obesity as numerous studies have established in adults, children and across ethnic groups [1], [2], [3].

However the causative factors for this association remain complex as obesity related hypertension is often associated with multiple concurrent metabolic abnormalities, including high levels of low-density lipoproteins (LDL), high triglycerides, high cholesterol, high fasting glucose, as well as low high-density lipoprotein (HDL) levels and diabetes mellitus type 2 (DMT2) [4], [5], [6]. Despite this, the locality of fat accumulation represents a significant factor to increase hypertension risk, noted by waist circumference and waist hip ratio measurement, which gives a more detailed insight into abdominal obesity, being even more strongly associated with hypertension risk than generalized obesity [7]. The influence of abdominal adipose tissue (AT) on hypertension risk may arise as this AT depot is considered to be particularly pathogenic in nature with increasing adiposity [8]. Previous work has also highlighted that AT is a significant point of action in the renin-angiotensin system (RAS) and through its effector hormone, angiotensin II (ANG II) may alter vasoconstrictive and pro-thrombotic properties associated with cardiovascular disease (CVD) [9], [10]. As such, it appears that as well as AT being an important site of RAS, abdominal obesity can specifically influence hypertension risk which may arise through the production of an array of hormones and cytokines, often referred to as ‘adipokines’ which may have direct and indirect effects on hypertension risk [11]. This suggests that AT action and dysfunction, with weight gain, may directly impact on hypertension. Adipose tissue dysfunction is characterized by adipocyte hypertrophy, increased macrophage recruitment, and changes in adipokine secretion profile resulting in a pro-inflammatory response. It can also influence hypertension risk particularly as previous studies have indicated the importance of adipokines in the potential regulation of blood pressure alongside insulin status [11], [12].

Tumor necrosis factor-alpha (TNF-α) is known to regulate angiotensinogen (AGT) in hepatocytes, as the AGT promoter contains a cytokine-inducible enhancer known as an acute phase response element [13]. TNF-α induces transcription of AGT via the transcription factor nuclear factor-κB (NFκB, which is a key factor in the regulation of numerous pro-inflammatory adipokines [12]. This concept has since been further supported by demonstrating that AT is a significant source of AGT and ANG II production; further that with increasing TNF-α release from venous drainage of the Sc AT, this is strongly correlated with ANG II secretion from the same source [11], [12]. Subsequent *in vitro* AT studies endorse the noted *in vivo* regulatory effect of TNF-α on both AGT and ANG II secretion [12]. Whilst the role of TNF-α in hypertension risk appears apparent, it should also be stressed that other adipokines such as leptin, IL6, resistin, plasminogen activator inhibitor 1 (PAI-1) and adiponectin may also have a role [14], [15], [16], [17]. Specifically adiponectin is associated with obesity related hypertension, as it can affect vascular tone, cellular proliferation and inflammation [16], [17], [18].

As such, the aims of this study were to determine whether adiposity and/or adipokines alone or as a ratio may represent useful biomarkers of hypertension risk in a Saudi cohort with and without hypertension and or DMT2. To date studies examining the Saudi population and hypertension risk have been limited, although hypertension remains a serious health problem in the country [2], [19].

## Materials and Methods

### Subjects

A total of 377 (192 males and 185 females) adult Saudi subjects, aged 40–60 years, participated in this cross sectional study. They were selected from the existing Biomarkers Screening in Riyadh Program (RIYADH Cohort), a capital-wide study composed of randomly selected individuals from different Primary Health Care Centers (PHCCs) in Riyadh, KSA. The study was carried out at the Biomarkers Research Program, King Saud University, Riyadh, KSA. A questionnaire focusing on demographic information and past medical history was given to all participating subjects. The inclusion criteria were: ≥18 years of age; BMI ≥18 Kg/m^2^ and completed medical history questionnaire. Detailed medical drug histories were taken on medications and subjects on medication known to treat blood pressure were excluded. In addition, individuals with acute co-morbidities that needed immediate medical attention were excluded from the study. Written consent was obtained from each subjects entered into the study. Ethical approval was granted by the Ethics Committee of the College of Science Research Center, King Saud University, Riyadh, KSA.

### Anthropometric measurements

Weight, height, blood pressure, hip and waist circumferences were measured following the standard procedures. The Holtain Khan abdominal caliper by Holtain Ltd (Crymych, UK) was used to measure sagital abdominal diameter (SAD) as previously described [20]. Body mass index (BMI) was calculated as weight/height^2^ (Kg/m^2^). Subjects were subdivided into four groups: healthy lean controls, obese normotensive, hypertensive obese and hypertensive obese DMT2 patients. Obese subjects were defined as BMI ≥30 Kg/m^2^ and normal weight subjects having BMI of 18–25 according to world health organization (WHO) criteria. Hypertension was defined as systolic BP ≥140 mmHg and/or diastolic BP ≥90 mmHg [21]. Patients with DMT2 were defined by the WHO criteria (fasting blood glucose level more than 126 mg/dl (≥7 mM/L) or a 2 hr postprandial plasma glucose level ≥200 mgdl (≥11.1 mM/L).

### Biochemical parameters

Serum fasting blood glucose (FBG), total cholesterol, triglycerides and HDL-cholesterol were determined enzymatically using a chemical analyzer Konelab (Thermo Konelab 20i). Concentrations of LDL-cholesterol were calculated using Friedwald's formula. Serum leptin (inter-assay CV 15% with intra-assay CV 7.9%), adiponectin (inter-assay CV 15% with intra-assay CV 5.6%), resistin (inter-assay CV 13% with intra-assay CV 6%), insulin, (inter-assay CV 14% with intra-assay CV 5.1%), TNF-α (inter-assay CV 4% with intra-assay CV 6%) and PAI-1 (inter-assay CV 10% with intra-assay CV 6%) were measured using Luminex Milliplex kits (Millipore Corporation, MA, USA). Serum ANG II (inter-assay CV 7% with intra-assay CV 4%) was measured using Human ANG II EIA kit (Phoenix pharmaceuticals, Belmont, CA, USA) and hs-CRP (inter-assay CV 11.6% with intra-assay CV 6%) was measured by human CRP ELISA kit (Immunodiagnostik AG, USA). Homeostasis model assessment of insulin resistance (HOMA-IR) was calculated as fasting insulin (µU/mL)×fasting glucose (mmol/L)/22.5.

### Statistical analysis

Data are expressed as mean ± SD. Non Gaussian variables are represented by Median (inter-quartile range). Analysis of co-variance (ANCOVA) was carried out among different groups, adjusted for the covariate age. The Spearman's rank correlation test was performed with systolic blood pressure as the dependent variable and was followed by partial correlation analyses controlling for BMI. Values of *P*<0.05 were considered significant. Data were analyzed using SPSS for windows Statistical Package for Social Sciences (version 11.5 SPSS Inc., Chicago, IL, USA).

## Results

### Anthropometric data and changes in lipid levels among groups

Demographic and clinical characteristic for studied subjects are presented in table 1. Hypertensive obese DMT2 patients had raised BMI (*p*<0.001), waist (*p*<0.001), hip circumference (*p*<0.001), sagittal abdominal diameter (SAD; *p*<0.001), systolic blood pressure (BP) (*p*<0.001), diastolic BP (*p*<0.001), and age (*p* = 0.002) compared with non diabetic (ND) normotensive controls. Similarly, obese hypertensive had significantly raised BMI (*p*<0.001), waist (*p*<0.001), hip circumference (*p*<0.001), SAD (*p*<0.01), systolic BP (*p*<0.001), diastolic BP (*p*<0.001) than normotensive ND controls (Table 1). Non hypertensive obese patients also had an increased BMI (*p*<0.001), waist (*p*<0.001), hip circumference (*p*<0.001) than normotensive ND controls.

**Table 1:** Demographic and Clinical Characteristic for Studied Subjects.

	Normotensive ND Control	Obese Normotensive ND	Obese Hypertensive ND	Obese Hypertensive with DMT2
**N (Male/Female)**	96 (29/67)	98 (50/48)	99 (63/36)	97 (53/45)
**Age**	47.9±5.1	46.1±5.0	48.6±6.1	50.8±6.0* # $
**BMI(kg/m^2^)**	22.9±2.1	33.7±4.2*	36.5±7.7* #	35.3±6.7*
**Waist(cm)**	85.4±13.1	95.9±19.6*	100.0±18.4*	102.1±21.4*
**Hips(cm)**	96.2±14.6	109.0±21.5*	111.3±21.3*	109.6±23.4*
**WHR**	0.89±0.12	0.88±0.14	0.91±0.17	0.94±0.18
**SAD(cm)**	21.8±10.4	24.1±5.3	26.4±10.8*	26.8±5.7*
**Systolic BP(mmHg)**	116.8±9.0	118.7±9.8	132.9±16.0* #	139.6±14.6* #
**Diastolic BP(mmHg)**	75.4±6.1	77.1±6.2	86.4±8.3* #	88.8±8.2* #
**Triglyceride (mmol/l)**	1.6±0.30	1.6±0.32	1.8±0.35	2.1±0.35* # $
**T. Cholesterol (mmol/l)**	5.3±1.0	5.1±1.0	5.4±1.1	5.7±1.2* #
**HDL(mmol/l)**	0.86±0.28	0.76±0.27	0.82±0.31	0.80±0.32
**LDL(mmol/l)**	3.6±0.92	3.5±0.72	3.7±1.0	3.9±1.1#
**Glucose(mmol/l)**	5.6±1.6	5.9±2.0	5.3±1.0	10.7±3.8* # $
**Insulin(uIU/ml)**	8.6±1.3	10.4±1.1	12.8±1.2*	13.1±1.0*
**HOMA-IR**	1.78 (0.91,3.0)	2.2 (1.6, 3.6)	2.8 (2.0, 4.0)	5.4 (3.1, 9.1)* # $
**Adiponectin (ug/ml)**	13.5±1.1	9.3±1.2*	11.8±1.0	11.3±1.2
**Leptin(ng/ml)**	13.3±2.6	29.6±2.7*	41.5±2.7*	34.2±2.2*
**Leptin/adiponectin ratio**	1.48±1.7	2.4±1.7*	3.1±1.9*	2.7±1.8*
**Resistin (ng/ml)**	21.3±2.0	19.5±1.7	23.7±2.0	24.7±2.7#
**TNF-α(pg/ml)**	5.4±0.78	5.2±0.71	7.8±0.80* #	6.8±0.95* #
**hsCRP (mg/ml)**	2.1±1.7	4.2±2.5*	4.4±3.1*	4.1±2.6*
**ANG II (ng/ml)**	0.93±0.23	0.89±0.24	1.07±0.30* #	1.21±0.26* # $

**Data represented by N(%); Mean ± Std.deviation.**

‘*’ **indicate group is significantly from the control.**

‘#’ **indicates group is significantly different from the obese.**

$ **- group is significantly different from the obese hypertensive.**

Hypertensive obese DMT2 patients had significantly increased triglyceride (*p*<0.001) and cholesterol (*p*<0.05) compared with normotensive ND controls. After age adjustment, the difference in cholesterol levels between obese hypertensive DMT2 and normotensive ND controls was no longer significant, whilst differences in systolic BP, diastolic BP, BMI, waist, hip circumference and SAD remained significant.

### Changes in glucose, insulin and adipocytokines levels among normotensive and hypertensive subjects groups

FBG (*p*<0.001), insulin (*p*<0.001), HOMA-IR (*p*<0.001), leptin (*p*<0.001), leptin/adiponectin ratio (*p*<0.001), CRP (*p*<0.05), TNF-α (*p*<0.001) and ANG II (*p*<0.001) levels were higher in hypertensive obese patients with DMT2 than healthy normotensive ND controls. Insulin (*p* = 0.001), leptin (*p*<0.001), leptin/adiponectin ratio (*p*<0.001), CRP (*p*<0.05), TNF-α (*p*<0.001) and ANG II (*p*<0.05) levels were increased in obese hypertensive patients compared with healthy normotensive ND controls. Serum adiponectin concentration was lower (*p*<0.01) while leptin (*p*<0.001), leptin/adiponectin ratio (*p*<0.05), and CRP (*p*<0.01) were significantly higher in non-hypertensive obese than in healthy normotensive ND controls.

### Association between blood pressure and biochemical parameters among normotensive and hypertensive subjects groups

**Figure 1:**
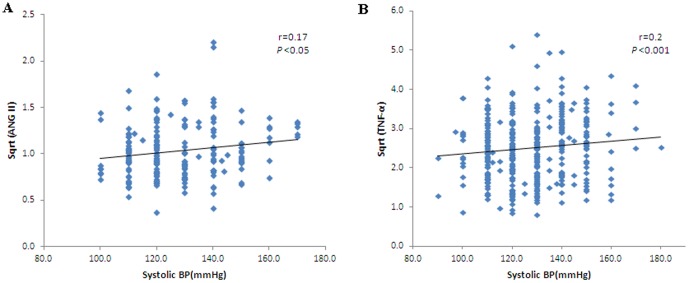
Correlation between systolic blood pressure and ANG II (r = 0.17, p<0.05) (A); TNF-α (r = 0.2, p<0.001) (B) in the entire group studied.

**Figure 2:**
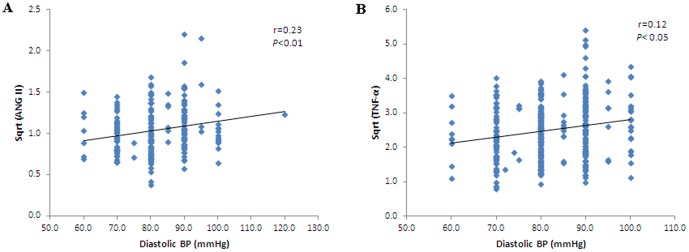
Correlation between diastolic blood pressure and ANG II (r = 0.2, p<0.01) (A); TNF-α (r = 0.12, p<0.05) (B) in the entire group studied.

Spearman correlations using systolic blood pressure as a dependent variable are shown in Table 2. Systolic BP was positively associated with BMI (r = 0.25, *p*<0.001), glucose (r = 0.257, *p*<0.001), insulin (r = 0.12, *p*<0.05), HOMA-IR (r = 0.24, *p*<0.001), leptin (0.153, *p*<0.01), ANG II (r = 0.17, *p*<0.05) (Fig. 1A), and TNF-α (r = 0.2, *p*<0.001) (Fig. 1B) across the combined cohort. Diastolic blood pressure was positively associated with BMI (r = 0.30, *p*<0.001), glucose (r = 0.19, *p*<0.001), insulin (r = 0.13, *p*<0.05), HOMA-IR (r = 0.27, *p*<0.001), leptin (0.14, *p*<0.01), ANG II (r = 0.23, *p*<0.01) (Fig. 2A), and TNF-α (r = 0.12, *p*<0.05) (Fig. 2B) across all groups.

**Table 2:** Spearman's Correlation Using Systolic BP, Diastolic BP, TNF-α and Angiotensin II as Dependent Variables.

	Systolic BP		Diastolic BP		Angiotensin II		TNF-α	
	r	P	r	P	r	P	r	P
**Waist/Hip ratio**	0.08	0.08	0.09	0.07	0.14	0.05	0.14	0.005
**BMI**	0.25	<0.001	0.30	<0.001	0.13	0.06	0.15	0.004
**SAD**	0.03	0.60	0.08	0.16	0.20	0.008	−0.02	0.78
**Glucose**	0.257	<0.001	0.1950	<0.001	0.114	0.100	0.099	0.059
**Insulin**	0.12	0.01	0.13	0.01	0.14	0.03	0.12	0.01
**HOMA-IR**	0.24	<0.001	0.27	<0.001	0.18	0.009	0.15	0.005
**Adiponectin**	−0.003	0.95	0.005	0.91	0.05	0.43	0.03	0.50
**Leptin**	0.153	0.004	0.148	0.006	0.17	0.01	0.38	<0.001
**Resistin**	0.04	0.39	0.03	0.57	0.13	0.04	0.09	0.08
**PAI-1**	0.08	0.12	0.07	0.18	0.07	0.33	0.23	<0.001
**CRP**	0.01	0.80	0.06	0.32	0.05	0.45	0.11	0.05
**TNF-α**	0.20	<0.001	0.12	0.01	0.32	<0.001	-	-
**ANG II**	0.17	0.01	0.23	0.001	-	-	0.32	<0.001

Spearman's correlation coefficient with corresponding p-value (p<0.05 is significant).

Using partial correlation analysis, controlling for BMI, positive associations between systolic BP and glucose (r = 0.243, *p*<0.001), insulin (r = 0.114, *p*<0.05), HOMA-IR (r = 0.22, p<0.001), TNF-α (r = 0.17, *p*<0.01) and ANG II (r = 0.13, *p*<0.05) remained significant across the combined cohort. In addition, using the same analysis, positive correlations were also noted between diastolic BP and glucose (r = 0.208, *p*<0.001), insulin (r = 0.13, *p*<0.05), HOMA-IR (r = 0.25, *p*<0.001), and ANG II (r = 0.13, *p*<0.01) across the combined cohort; whilst association with TNF-α was no longer significant.

### Association of ANG II and TNF-α with anthropometric and biochemical parameters among normotensive and hypertensive subjects

**Figure 3:**
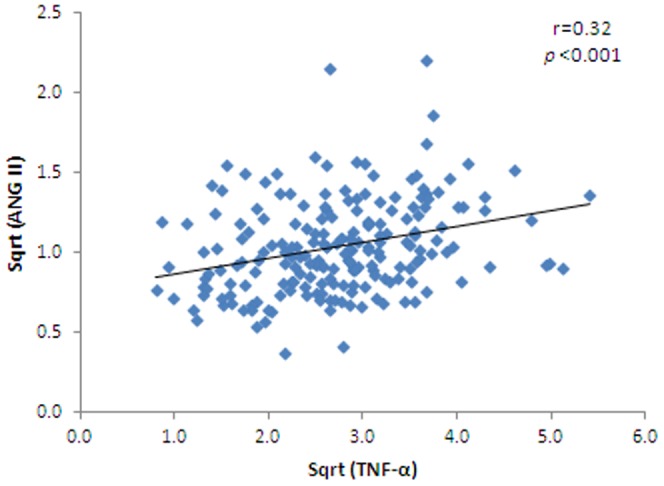
Correlation between ANG II and TNF-α (r = 0.32, p<0.001) in the entire group studied.

**Figure 4:**
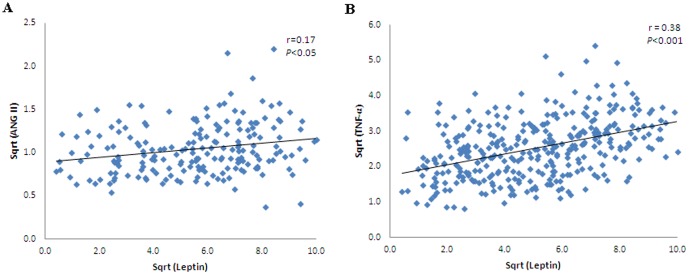
Correlation for leptin with ANG II (r = 0.17, *p*<0.05) (A); and TNF-α (r = 0.38, *p*<0.001) (B) in the entire group studied.

ANG II was positively associated with SAD (r = 0.2, *p*<0.01), insulin (r = 0.14, *p*<0.05), HOMA-IR (r = 0.18, *p*<0.01), TNF-α (r = 0.32, *p*<0.001) (Fig. 3), leptin (r = 0.17, *p*<0.05) (Fig. 4A), resistin (r = 0.13, *p*<0.05). TNF-α was positively associated with WHR (r = 0.14, *p*<0.01), BMI (r = 0.15, *p*<0.01), insulin (r = 0.12, *p*<0.05), HOMA-IR (r = 0.15, *p*<0.01), leptin (r = 0.38, p<0.001) (Fig. 4B), PAI-1(r = 0.23, *p*<0.001), and ANG II (r = 0.32, *p*<0.001) (Table 2).

Using partial correlation analysis controlling for BMI, significant positive associations between ANG II and TNF-α (r = 0.22, *p* = 0.001), remained significant across the combined cohort, whilst associations between ANG II and SAD, insulin , HOMA-IR, leptin and resistin were no longer significant (Table 3).

**Table 3:** Partial Correlation Using Systolic BP, Diastolic BP, TNF-α and Angiotensin II as Dependent Variables Controlled for BMI.

	Systolic BP		Diastolic BP		Angiotensin II		TNF-α	
	r	*p*	r	*p*	r	*p*	r	*p*
**Waist/Hip ratio**	0.078	0.206	0.106	0.04	0.157	0.025	−0.143	0.007
**SAD**	−0.041	0.508	0.009	0.88	0.003	0.967	−0.112	0.078
**Glucose**	0.243	<0.001	0.208	<0.001	0.058	0.409	0.027	0.613
**Insulin**	0.114	0.032	0.13	0.01	0.068	0.342	0.046	0.391
**HOMA-IR**	0.222	<0.001	0.25	<0.001	0.109	0.129	0.040	0.451
**Adiponectin**	0.043	0.425	0.039	0.463	0.046	0.523	0.051	0.347
**Leptin**	0.011	0.839	−0.007	0.899	0.142	0.05	0.346	<0.001
**Resistin**	0.009	0.870	−0.008	0.886	0.103	0.143	0.073	0.172
**PAI-1**	0.075	0.181	0.061	0.279	0.140	0.057	0.012	0.832
**CRP**	−0.118	0.05	−0.078	0.207	−0.078	0.279	−0.009	0.886
**TNF-α**	0.170	0.001	0.083	0.115	0.229	0.001	-	-
**ANG II**	0.131	0.039	0.196	0.005	-	-	0.229	0.001

Spearman's correlation coefficient with corresponding p-value (p<0.05 is significant).

Using partial correlation analysis, controlling for BMI, positive correlations between TNF-α, WHR (r = 0.14, *p*<0.01), leptin (r = 0.34, *p*<0.001), and ANG II (r = 0.22, *p*<0.01) remained significant, whilst association between TNF-α and insulin, HOMA-IR, PAI-1 were no longer significant (Table 3).

## Discussion

Despite the high prevalence of obesity and hypertension in Saudi Arabia, to date, no study has examined associations between adipokines and BP in the obese hypertensive phenotype. Furthermore as obesity, DMT2 and hypertension are increasingly considered to develop through sub-clinical chronic inflammation, the role of the pro-inflammatory adipokines becomes increasingly more important to understand [22]. From these current studies ANG II and TNF-α were identified as key biomarkers of hypertension risk in the Saudi cohorts, which remained significant independent of DMT2, age or BMI. Specifically, both systolic and diastolic blood pressure, were noted to remain positively associated with TNF-α and ANG II across the combined cohort. This indicates the clear impact of these pro-inflammatory factors systemically. However it should also be stressed that other inflammatory factors, such as leptin, leptin/adiponectin ratio and CRP, were also identified to be increased in the obese hypertensive with and without DMT2 compared with the normotensive ND controls.

The concept that pro-inflammatory adipokines are important in hypertension risk may not necessarily be surprising since previous studies have shown that other inflammatory markers, such as CRP are increased in patients with obesity, DMT2 and hypertension, as well as predict the development of these diseases [22], [23], [24]. Furthermore AT derived ANG II drains directly into the circulation and is known to affect vascular pathophysiology being able to trigger vascular inflammation as well as oxidative stress [12], [25], [26]. In this mechanism, ANG II can mediate changes in transcription through NFκB leading to an increase in inflammatory cytokines and reactive oxygen species, all factors that induce vascular injury [25], [27].

This current study further revealed positive correlations between BP and serum levels of TNF-α along with ANG II. Such a positive relationship between BP and TNF-α has been noted in previous studies [28], [29]. This finding, therefore, further supports the concept that increased BP is associated with vascular inflammation [30] particularly as TNF-α decreases nitric oxide (NO) production and enhances NO removal in endothelial cells leading to impaired NO-mediated vasodilation [31], [32]. Also similar to ANG II, TNF-α induces the production of ROS providing one mechanism for NO depletion [33]. TNF-α may affect BP also indirectly via the RAS, as it has been shown to increase production of AGT, ANG II type 1 receptors and ANG II [34]. As inflammation is a key component in the pathogenesis of hypertension, the interaction between ANG II and TNF-α may play an important role in the modulation of hypertensive response [35]; ANG II treatment induces the production of TNF-α in cultured cardiomyocytes and fibroblasts [36]. Consistently our results showed a positive association between TNF-α and ANG II which could explain the link between vascular inflammation and hypertension via the elevation of both TNF-α and ANG II levels

Further biochemical analysis showed that with a hypertension phenotype, with and without DMT2, there was a positive association with adipokines such as leptin, leptin/adiponectin ratio, as well as TNF-α and ANG II. The role of leptin in hypertension has been previously detailed with leptin-mediated increases in BP being prevented by adrenergic blockade [37], [38]. Also leptin deficient mice have normal or reduced BP, despite severe obesity, suggesting that a functional leptin system may be necessary for obesity to increase sympathetic nerve activity and BP [37], [38], [39]. In humans, further data highlights a positive relationship between hyperleptinaemia and hypertension in both men and women, and this effect may be independent of BMI and insulin resistance [14], [40], [41]. Furthermore studies also suggest a potential interaction between leptin and the RAS as the use of ANG-converting enzyme inhibitors and ANG receptor blockers leads to a reduction in circulating leptin levels, which is associated with improved blood pressure control [42], [43]. Leptin also acts as a pro-inflammatory factor and therefore may have a direct impact on the vasculature, similar to ANG II and TNF-α.

Our studies also showed that the leptin/adiponectin ratio was associated with the hypertension phenotype, indicating that high leptin and low adiponectin would increase the likelihood of a subject being hypertensive. Previous studies have shown that hypoadiponectinemia is reported to be a risk factor for hypertension and associated with endothelial dysfunction [44], [45]. Several cross-sectional studies have previously shown an inverse relationship between adiponectin levels with both systolic and diastolic BP [46], [47]. In contrast, our study did not note any significant association between adiponectin and BP, assessed alone, as matched from the Anglo-Scandinavian Cardiac Outcomes Trial (ASCOT) study [48]. In the case of leptin an association remained with a hypertension phenotype which suggests that leptin has a more significant influence than adiponectin in this Saudi cohort. To date, there is conflicting evidence on the importance of leptin and adiponectin, either as a ratio or alone, in determining hypertension risk; such conflict may arise due to multiple confounders which ultimately affect both circulating leptin and adiponectin levels [48], [49], [50].

Finally, our anthropometric data affirms previous studies that a hypertension phenotype, with and without DMT2, is associated with raised BMI, waist, hip circumference and SAD which remains post age adjustment. This highlighted that the Saudi cohorts were similar to other ethnicities in identifying that obesity and more specifically abdominal obesity are positively associated with increased BP as noted by both systolic and diastolic BP rises [51], [52].

In conclusion our data suggest that circulating ANG II and TNF-α are associated with a hypertension phenotype independent of age, diabetic status and BMI, being positively associated with arterial BP. In addition that both abdominal adiposity and diabetic status can exacerbate hypertension risk, in line with previous studies. As such AT appears to represent an important site of ANG II and TNF-α production. Taken together these adipokines may therefore represent important biomarkers to evaluate hypertension risk, as well as provide a mechanistic insight into the pathogenesis of obesity-related hypertension.
